# Supplementary material for: A centrally positioned cluster of multiple centrioles in antigen-presenting cells fosters T cell activation
Source: Nat Commun. 2026 Jan 13;17:536. doi: 10.1038/s41467-026-68286-7 (PMC12804990; doi:10.1038/s41467-026-68286-7)
Supplement: Supplementary file 1 — Supplementary Information [file 41467_2026_68286_MOESM1_ESM.pdf]

Supplementary Information for

A centrally positioned cluster of multiple centrioles in antigen-presenting cells fosters T cell activation

Isabel Stötzel, Ann-Kathrin Weier, Apurba Sarkar, Subhendu Som, Luisa Bach, Peter Konopka, Eliška Miková, Shaunak Ghosh, Jan Böthling, Mirka Homrich, Laura Schaedel, Uli Kazmaier, Konstantinos Symeonidis, Stefan Ebner, Philip Weidner, Zeinab Abdullah, Felix Meissner, Stefan Uderhardt, Miroslav Hons, Dirk Baumjohann, Raja Paul, Heiko Rieger*, Eva Kiermaier*

*Eva Kiermaier
Email: eva.kiermaier@uni-bonn.de

*Heiko Rieger
Email: heiko.rieger@uni-saarland.de

This PDF file includes:

Supplementary Methods
Supplementary Figures 1 to 11
Supplementary Table 1
SI References

Other supplementary materials for this manuscript include the following:

Supplementary Movies 1 to 8

Supplementary Methods

Analytical prediction of optimal centrosome position in the absence of a nucleus

We find the optimal position of the centrosome (or centriole cluster), corresponding to the minimum average geometric distance between the centrosome and the cell surface points, inside a 2*D* circular and 3*D* spherical cell of radius R_{cell} without a nucleus.

In 2*D* **circular cell:** In the Cartesian coordinate frame, consider the position of centrosome (x_0, y_0) and the cell center at the origin $(0, 0)$. Then, the distance between an arbitrary point (x, y) on the cell surface and the centrosome is

$$d^2 = (x - x_0)^2 + (y - y_0)^2$$

$$= (R_{cell} cos\theta - x_0)^2 + (R_{cell} sin\theta - y_0)^2$$

Here, $R_{cell} cos\theta$ and $R_{cell} sin\theta$ represent the parametric equations of x and y, respectively, in the plane polar coordinate system. θ $(\in 0 - 360^0)$ is the counterclockwise angle (also called polar angle) measured with respect to the positive x-axis of the cell. Therefore, the average distance relative to the entire cell surface would be

$$< d^2 >= \frac{1}{2\pi} \int_0^{2\pi} (R_{cell} cos\theta - x_0)^2 + (R_{cell} sin\theta - y_0)^2 \, d\varphi$$

$$= \frac{1}{2\pi} \int_0^{2\pi} d\varphi \left\{ R_{cell}^2 (cos^2\theta + sin^2\theta) - 2R_{cell} x_0 cos\varphi - 2R_{cell} y_0 sin\varphi + (x_0^2 + y_0^2) \right\}$$

$$= R_{cell}^2 + x_0^2 + y_0^2$$

Therefore,

$$< d^2 >_{min} = R_{cell}^2 \text{ with } x_{0,min} = 0, y_{0,min} = 0$$

$$< d^2 >_{max} = 2R_{cell}^2 \text{ with } x_{0,max}, y_{0,max} \in \text{points on the cell surface}$$

Therefore, the minimum of the average geometric distance, $< d_{short} >$ $(= \sqrt{< d^2 >_{min}} = R_{cell}$), correspond to the optimal centrosome position at the center of the circular cell.

In 3*D* **spherical cell:** Similar to above, the distance between an arbitrary 3*D* point (x, y, z) on the surface of a spherical cell (center is at $(0, 0, 0)$) and the centrosome location (x_0, y_0, z_0) follows

$$d^2 = (x - x_0)^2 + (y - y_0)^2 + (z - z_0)^2$$

$$= (R_{cell} sin\theta cos\varphi - x_0)^2 + (R_{cell} sin\theta sin\varphi - y_0)^2 + (R_{cell} cos\theta - z_0)^2$$

Here, $R_{cell} sin\theta cos\varphi$, $R_{cell} sin\theta sin\varphi$, and $R_{cell} cos\theta$ represent the parametric equation of x, y, and z, respectively, in a spherical polar coordinate system. θ $(\in 0 - 180^0)$ and φ $(\in 0 - 360^0)$ denote the polar and azimuthal angle, measured with respect to the positive *z* and *x* axes of the cell, respectively.

Therefore, the average distance relative to the entire cell surface would be

$$< d^2 > = \frac{1}{4\pi}\int_0^{2\pi} d\varphi \int_0^{\pi} d\theta sin\theta \, (x - x_0)^2 + (y - y_0)^2 + (z - z_0)^2$$

$$= \frac{1}{4\pi}\int_0^{2\pi} d\varphi \int_0^{\pi} d\theta sin\theta \, \{(x^2 + y^2 + z^2) + (x_0^2 + y_0^2 + z_0^2) - 2(xx_0 + yy_0 + zz_0)\}$$

$$= R_{cell}^2 + (x_0^2 + y_0^2 + z_0^2)$$

$$- \frac{1}{2\pi}\int_0^{2\pi} d\varphi \int_0^{\pi} d\theta sin\theta \, R_{cell}(x_0 sin\theta cos\varphi + y_0 sin\theta sin\varphi + z_0 cos\theta)$$

$$= R_{cell}^2 + x_0^2 + y_0^2 + z_0^2$$

Therefore,

$< d^2 >_{min} = R_{cell}^2$ with $x_{0,min} = 0, y_{0,min} = 0, z_{0,min} = 0$

$< d^2 >_{max} = 2R_{cell}^2$ with $x_{0,max}, y_{0,max}, z_{0,max}$ $\in$ points on the cell surface

Therefore, the minimum of the average geometric distance, $< d_{short} >$ ($= \sqrt{< d^2 >_{min}} = R_{cell}$), correspond to the optimal centrosome position at the center of the spherical cell.

Mathematical estimation of average search time in the absence of a nucleus predicts enhanced T cell priming capacity with increased MT numbers

Efficient MT docking at the IS in T cells has recently been described by a mathematical model[1]. The average search time of dynamic MTs in DCs can be estimated within this model framework. For a spherical cell of radius $R_{cell} = 18$ µm and without a nucleus, the average distance between the optimally localized centrosome (or centriole cluster) at the cell center and an arbitrary point on the cell boundary is 18 µm. For a MT growing from the centrosome with a velocity $\sim$ 15 µm/min (Holy et al., 1994)[2] needs around 72 sec to reach the cell boundary without undergoing any catastrophe. In case the MT hits the IS it docks, in case it does not hit the IS it shrinks again with approximately the same velocity as it grows, which means that an unsuccessful growth attempt (i.e. not hitting the IS) needs approximately $\tau_{trial} = 144$ sec.

The probability, p_{dock}, for a single growing MT to dock at a single target IS, is equal to the ratio of the target (IS) area and the total cell area: $p_{dock} = \frac{A_\tau}{A_{cell}} = \frac{\pi R_\tau^2}{4\pi R_{cell}^2} \approx 0.0031$ for an IS radius of $R_\tau = 2$ µm (Brossard et al., 2005)[3]. Consequently, a single MT needs on average $1/p_{dock} \approx 323$ search trials to dock at a single IS. In case of n synapses, the number of trials is reduced by a factor of $1/n$ since the total area of n synapses is simply n times the area of one IS. DCs contain between $N_{MT} \approx 35$ and 45 dynamic MTs during IS formation performing simultaneous search. The probability that at least one of

N_{MT} simultaneously growing MTs docks at a single IS is derived as follows: the probability that a single growing MT does *not* dock at the IS is $(1 - p_{dock})$, hence the probability that N_{MT} growing MTs do *not* dock at the IS is $(1 - p_{dock})^{N_{MT}}$, and therefore the probability that at least one MT docks is $1 - (1 - p_{dock})^{N_{MT}} = N_{MT}p_{dock} + \frac{1}{2}\, N_{MT}(N_{MT} - 1)p_{dock}^{2} + \ldots$. When $N_{MT} \cdot p_{dock}$ is much smaller than 1, one can neglect the terms following $N_{MT} \cdot p_{dock}$. Therefore, the probability that among N_{MT} dynamic MTs performing a simultaneous search, at least one docks at the IS, is simply N_{MT} times the probability that a single MT docks. Note that for large N_{MT}, i.e. when $N_{MT}p_{dock}$ is of order 1, one cannot neglect terms of higher order in $N_{MT}p_{dock}$ any more, the correct expression for the probability then being $1 - (1 - p_{dock})^{N_{MT}}$, which is still monotonously increasing with the number of MTs, N_{MT}.

The docking probability can immediately be translated into the average time that a dynamic MT needs to dock at a single IS: one unsuccessful growth-shrinkage trial lasts on average $\tau_{trial} = 144$ sec and on average a single MT needs $1/p_{dock} = 323$ trials to dock. Thus, a single MT needs on average $\tau_{trial} \cdot \frac{1}{p_{dock}} \approx 775$ min to dock at a single IS, and N_{MT} MTs need $1/(N_{MT}\, p_{dock})$ trials, i.e. only a fraction of $1/N_{MT}$ of the time for a single MT: 35/40 MTs need on average $\sim 22/19$ min to dock at a single IS, and 45/50 MTs need $\sim 17/15$ min. An immediate prediction of these considerations is that the docking efficiency of MTs increases with increasing numbers of MTs performing search and capture dynamics, which in turn increases T cell priming capacity.

Supplementary Figure 1

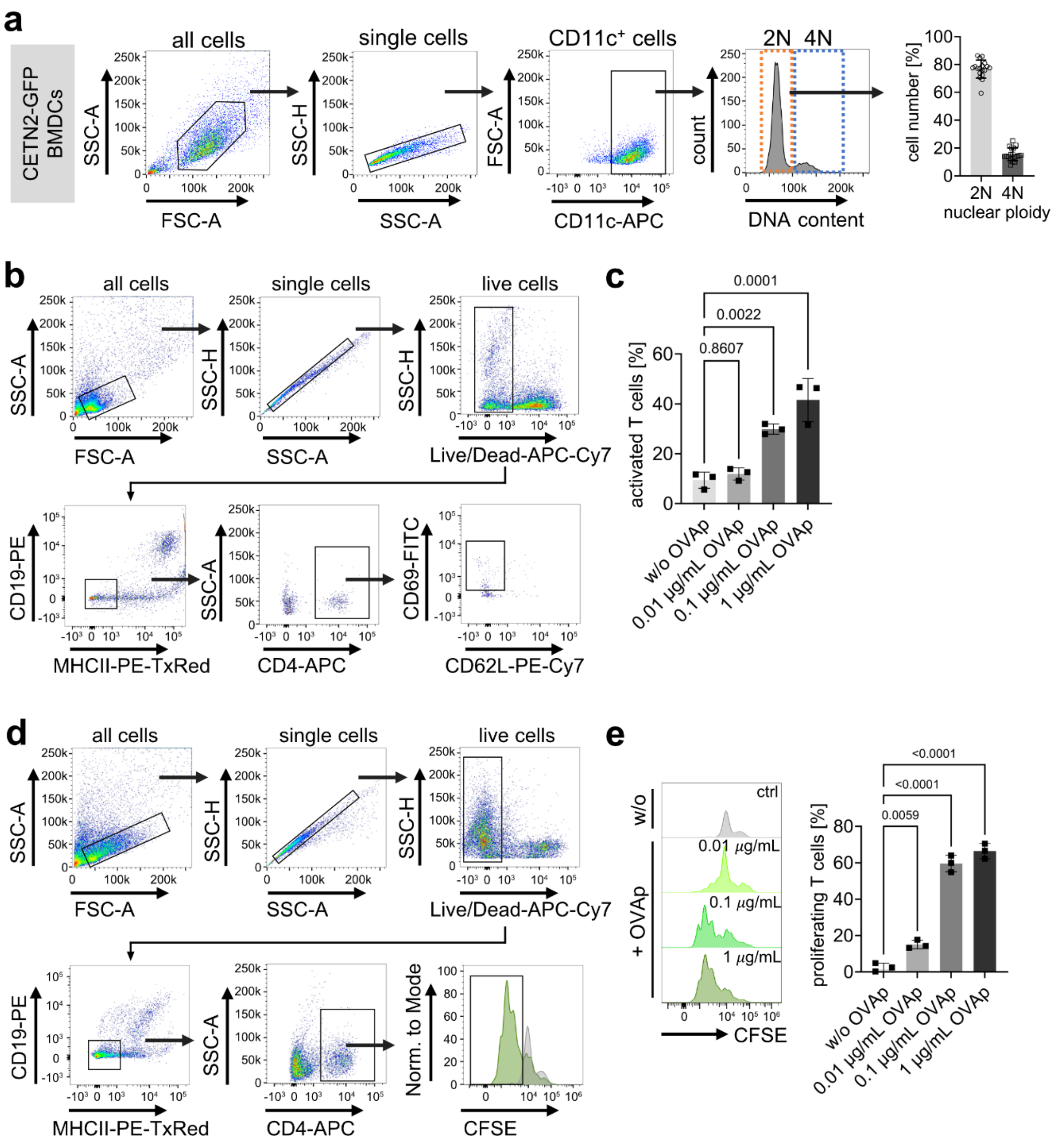

Supplementary Fig. 1.

Sorted diploid BMDCs induce T cell activation and proliferation. (**a**) DNA staining of mature CETN2-GFP expressing BMDCs to determine nuclear ploidy. Gating strategy for identification of 2N and 4N DCs and histogram of DNA content distribution of $CD11c^{+}$ cells. Graph displays mean values ± s.d. of 18 independent experiments. (**b**) Gating strategy for analyzing $CD69^{+}/CD62L^{-}$ activated $CD4^{+}$ T cells in the absence of antigen (w/o OVAp) or in the presence of different concentrations of OVAp. (**c**) Quantification of antigen-specific T cell activation. Graph shows mean values ± s.d. of three technical replicates of one out of four independent experiments. P values from one-way Anova with Dunnett's multiple comparisons. (**d**) Gating strategy for quantifying T cell proliferation in the absence of antigen (w/o OVAp) or in the presence of different concentrations of OVAp. (**e**) Quantification of antigen-specific T cell proliferation. Histograms show CFSE signal of $CD4^{+}$ T cells for different OVAp concentrations. Graph shows mean values ± s.d. of three technical replicates of one out of four independent experiments. P values from one-way Anova with Dunnett's multiple comparisons. (**a-e**) *N* = 10.000 cells per condition. Source data are provided as a Source Data file. Norm.: normalized, w/o: without.

Supplementary Figure 2

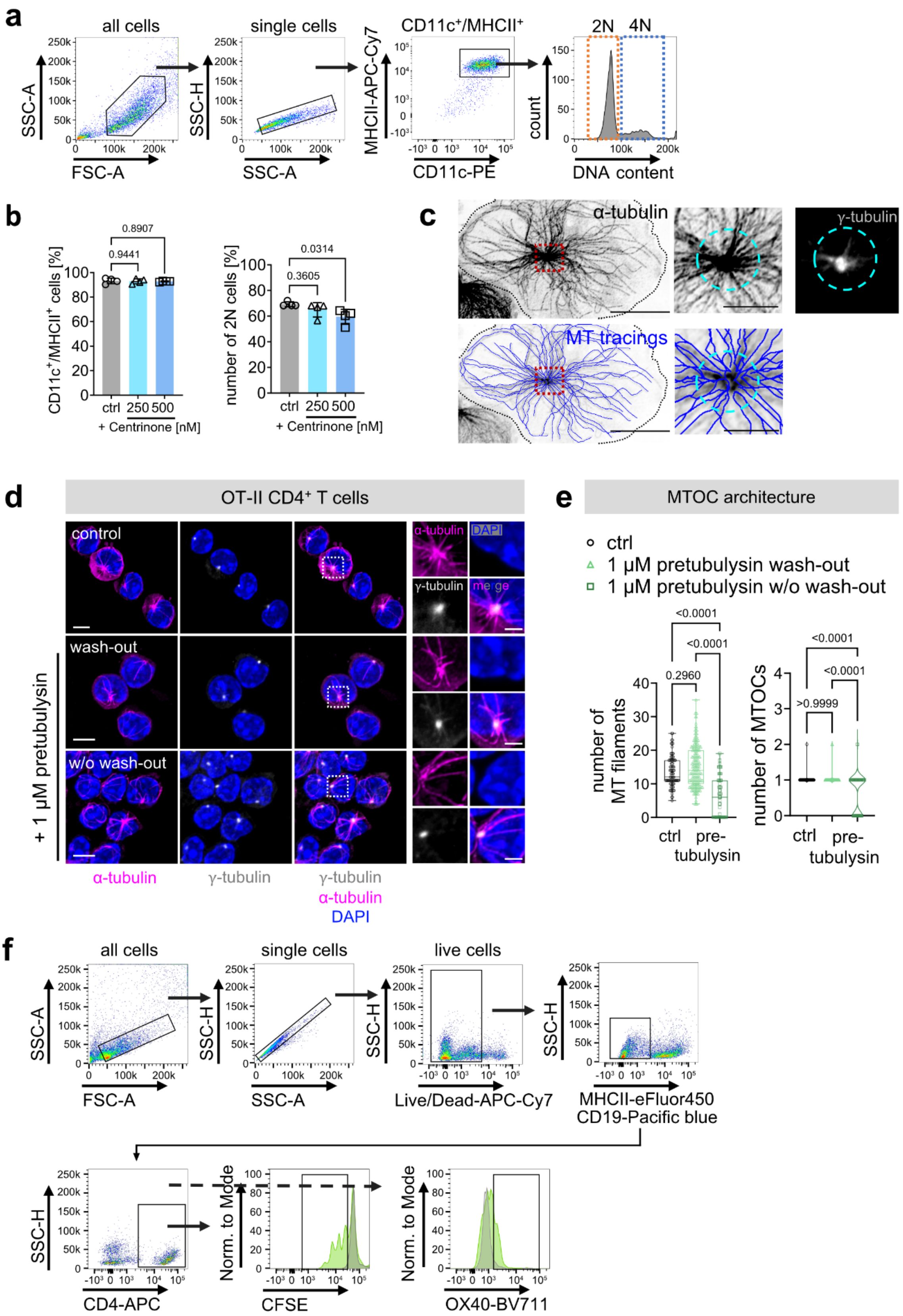

Supplementary Fig. 2.

Centrioles and an intact MT array are important for optimal T cell priming. (**a**) Gating strategy to assess DC differentiation in Centrinone-treated and control cells. Mature DCs were identified as $MHCII^{+}/CD11c^{+}$ cells and further analyzed for DNA content. (**b**) Quantification of $MHCII^{+}/CD11c^{+}$ (left graph) and 2N (right graph) cells in Centrinone-treated and control cells. Graphs show mean values ± s.d. of 4 independent experiments with cells derived from 4 different mice. *N* = 10.000 cells analyzed per condition. P values from one-way Anova with Dunnett's multiple comparisons. (**c**) Illustration of MTOC and MT number analysis in DCs stained with α-tubulin (grey-inverted) and γ-tubulin (white). MTOCs were defined as MT nucleation sites with visible γ-$tubulin^{+}$ foci. MT filaments were counted within the indicated ROI (cyan circle, area: 25 μm²). Tracings of MTs (blue) are shown below. Maximum z-projections are displayed. For MT counting, whole confocal stacks were used. Scale bars, 10 μm. In magnified insets, 5 μm. (**d**) Immunostaining of T cells co-cultured for 24h with BMDCs previously treated with pretubulysin according to (Fig. 2d). Maximum z-projections of merged and individual channels of α-tubulin (magenta), γ-tubulin (white) and DAPI (blue) are shown. Scale bars, 10 μm. Indicated regions are shown magnified (right panel). Scale bars, 2 μm. (**e**) Quantification of MT filaments (left) and MTOCs (right) in T cells. Left graph shows median, interquartile range and minimum to maximum values of four independent experiments. Each data point represents one cell. *N* = 93 (ctrl) /183 (pretubulysin wash-out) /60 (pretubulysin w/o wash-out). Right graph shows median and distribution of data points of four independent experiments. *N* = 103 (ctrl) /184 (pretubulysin wash-out) /61 (pretubulysin w/o wash-out). P values from Kruskal-Wallis test with Dunn's multiple comparisons. (**f**) Gating strategy to assess OX40 expression and proliferation of $CD4^{+}$ T cells after co-culture with pretubulysin-treated BMDCs. Grey filled lines in histograms represent condition w/o OVAp; green filled line with 0.1 μg/mL OVAp. Source data are provided as a Source Data file. ctrl: control, Norm.: normalized.

Supplementary Figure 3

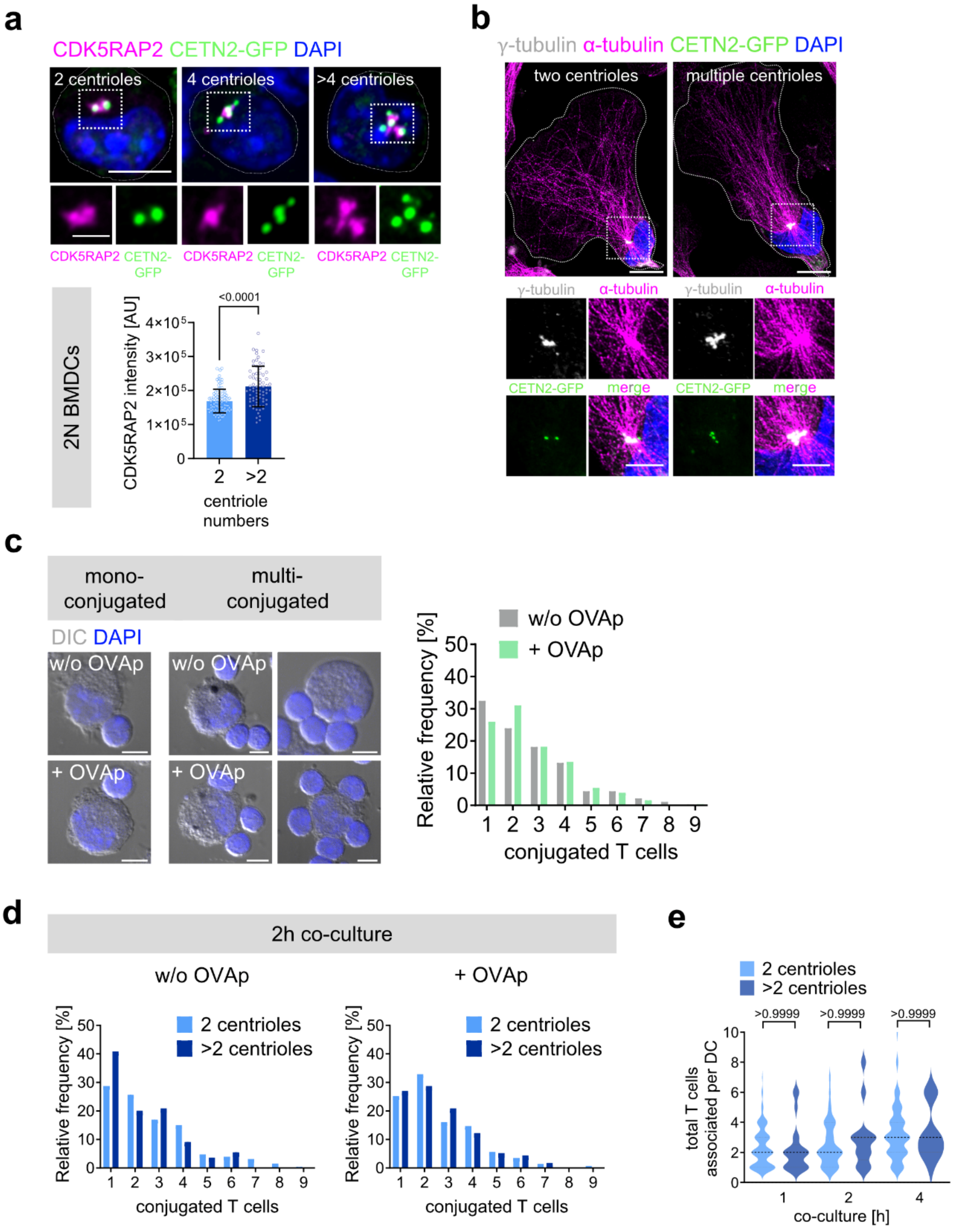

Supplementary Fig. 3.

IS formation in the presence of multiple centrioles. (**a)** Immunostaining of CDK5RAP2 in sorted 2N mature CETN2-GFP BMDCs. Merged channels of CDK5RAP2 (magenta), CETN2-GFP (green) and DAPI (blue) are shown. Scale bar, 5 μm. Insets show magnification of indicated regions. Individual channels of CETN2-GFP (green) and CDK5RAP2 (magenta) are shown. Scale bars, 2 μm. Below: Quantification of CDK5RAP2 signal intensity surrounding the centrioles in DCs. Graph shows mean values ± s.d. of one out of three independent experiments. Each data point represents one cell. *N* = 98 (2 centrioles)/74 (>2 centrioles). P value from two-tailed Mann-Whitney test. (**b**) Immunostaining of MT filaments in sorted 2N mature CETN2-GFP expressing BMDCs without T cells. White dotted boxes indicate magnified regions below. Merged and individual channels of CETN2-GFP (green), γ-tubulin (white) and α-tubulin (magenta) are shown. Cells were counterstained with DAPI (blue). Scale bars, 10 μm (upper panels) and 2 μm (insets bottom). (**c**) Left: Merged channels of differential interference contrast (DIC, grey) and DAPI (blue) of DC-T cell conjugates after 2h co-culture without (upper panels) or with (lower panels) previous OVAp loading. Scale bars, 5 μm. Right: quantification of frequency distribution of bound T cells per DC. Graphs display normalized values ± s.d. *N* = 397 (w/o OVAp) /225 (+ OVAp). (**d**) Histogram of frequency distribution of bound T cells to a single DC after 2 h of conjugate formation in cells with two (light blue) and >2 (dark blue) centrioles. w/o OVAp: *N* = 254/110 (2/>2 centrioles). + OVAp: *N* = 143/115 (2/>2 centrioles). (**e**) Quantification of bound T cells per DC after 1, 2 and 4 h of conjugate formation. Graph shows median and distribution of values of 5 independent experiments with *N* = 132 (1h) /59 (2h)/ 97 (4h) cells analyzed. P values from Kruskal-Wallis test with Dunn's multiple comparisons. (**c-e**) All images represent maximum z-projections. Source data are provided as a Source Data file. w/o: without.

Supplementary Figure 4

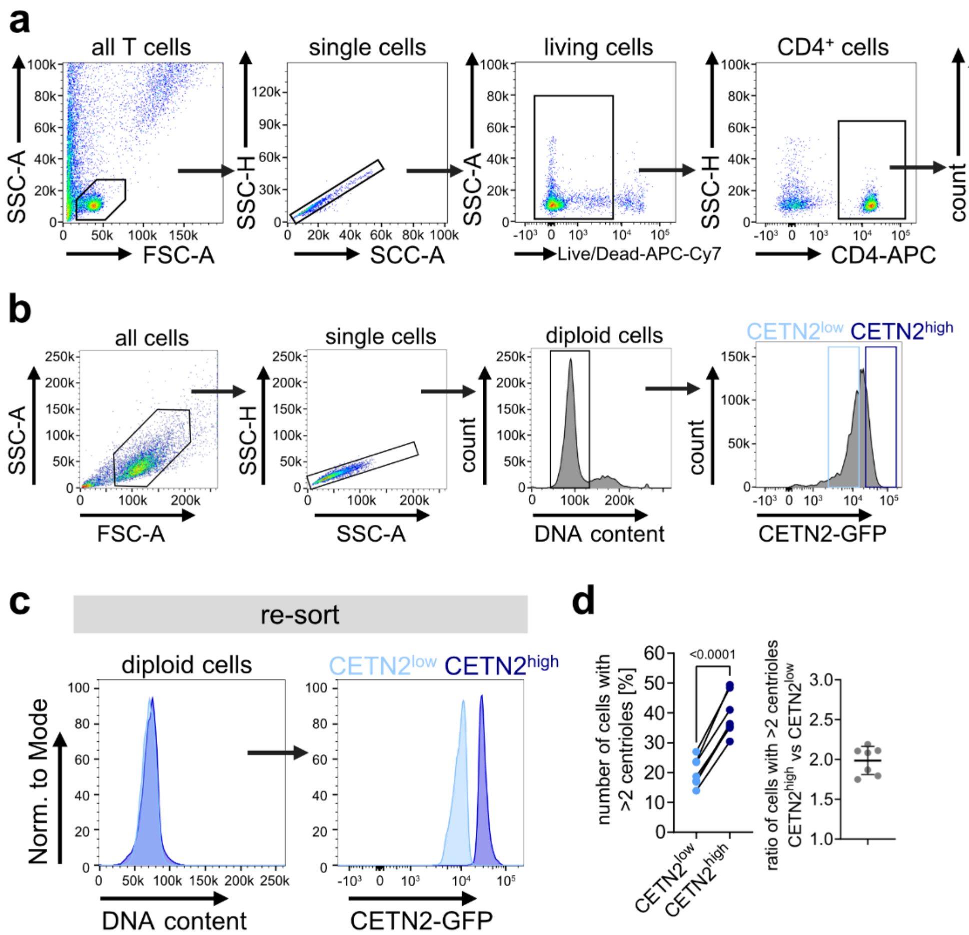

Supplementary Fig. 4.

Enrichment of DCs containing multiple centrioles. (**a**) Gating strategy for analyzing Nur77^{GFP} expression in CD4^{+} T cells. T cells without DC co-culture served as control and are included as light grey filled line. (**b**) Separation of CETN2-GFP^{low} (light blue) and CETN2-GFP^{high} (dark blue) expressing mature BMDCs. Single cells were gated on 2N and further separated according to CETN2-GFP signal intensities into CETN2-GFP high and low expressing cells. (**c**) Post-sort analysis of CETN2-GFP^{low} (light blue) and CETN2-GFP^{high} (dark blue) expressing cells for DNA content (left) and CETN2-GFP signal intensities (right). (**d**) Left: quantification of percentage of cells with >2 centrioles in CETN2-GFP^{low} (light blue) and CETN2-GFP^{high} (dark blue) expressing cells. Centriole numbers of sorted DC subpopulations were determined by confocal microscopy according to CETN2-GFP/γ-tubulin^{+} foci. Each data point represents one independent experiment (n = 7). CETN2-GFP^{low} N = 219/253/238/263/223/226/213; CETN2-GFP^{high}: N = 220/253/176/254/207/234/236. P value from two-tailed, paired Student's t-test. Right: ratio of cells with multiple centrioles between CETN2-GFP^{high} and CETN2-GFP^{low} cells. Source data are provided as a Source Data file. Norm.: normalized.

Supplementary Figure 5

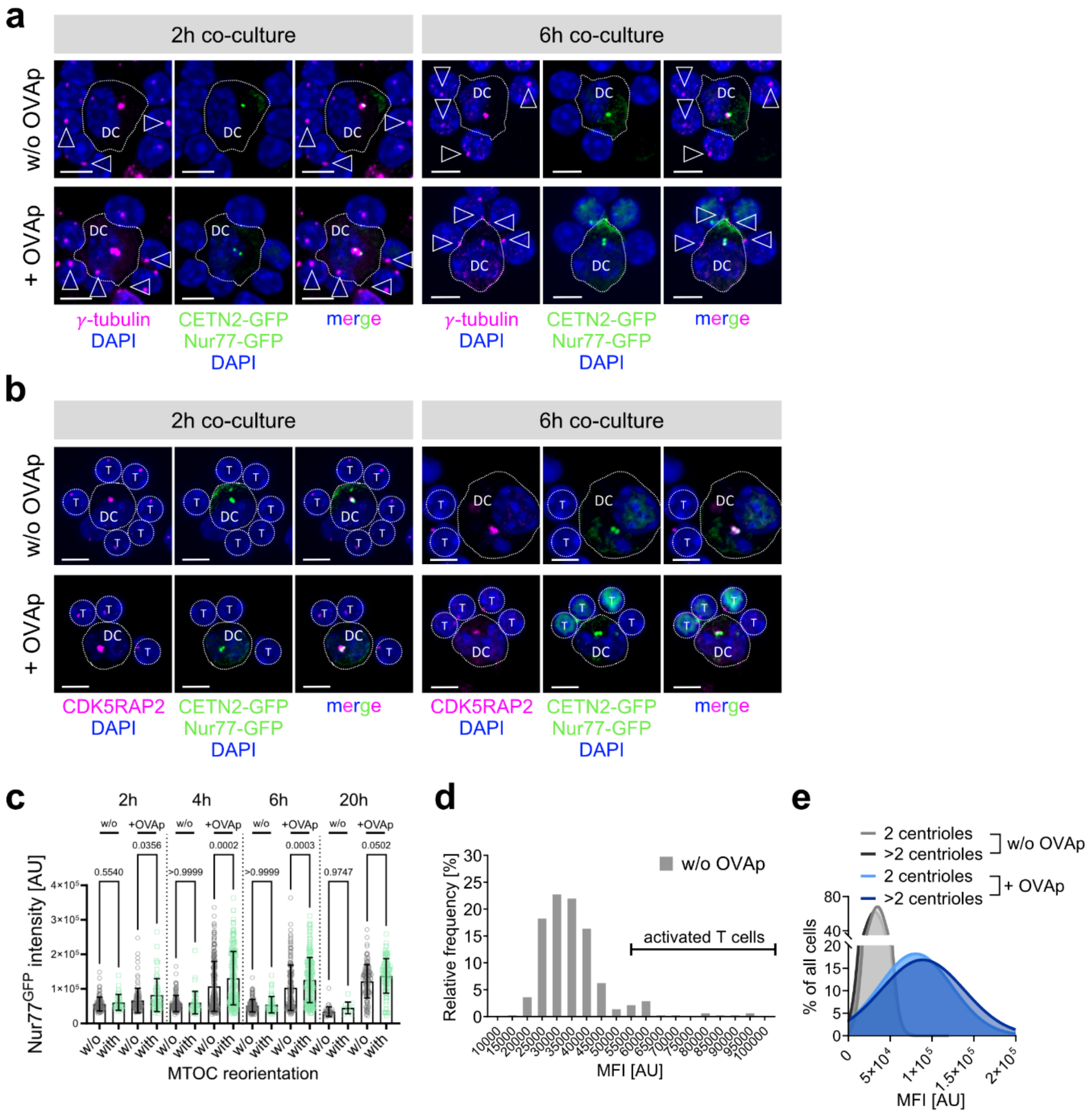

Supplementary Fig. 5.

Enhanced T cell activation in the presence of multiple centrioles in DCs. (**a**) Immunostaining of γ-tubulin (magenta) in conjugates of CETN2-GFP BMDCs and $Nur77^{GFP}$/OT-II $CD4^{+}$ T cells. Merged and individual channels of γ-tubulin (magenta), CETN2-GFP (green), $Nur77^{GFP}$ (green) and DAPI (blue) are shown. DC outline is indicated with dotted line. White arrowheads point to the T cell's centrosome. Scale bars, 5 μm. (**b**) Immunostaining of CDK5RAP2 (magenta) in conjugates of CETN2-GFP BMDCs and $Nur77^{GFP}$/OT-II $CD4^{+}$ T cells. Nuclei were counterstained with DAPI. Merged and individual channels of CDK5RAP2 (magenta), CETN2-GFP (green), $Nur77^{GFP}$ (green) and DAPI (blue) are shown. Dotted lines indicate DC outline; round circles the areas of GFP measurements. Scale bars, 5 μm. (**c**) Quantification of GFP signal intensities in T cells from microscopic images in the presence and absence of antigen and in dependence of MTOC reorientation in the T cell towards the IS. Graph shows one representative experiment out of three independent experiments. N = 333(2 h)/625(4 h)/584(6 h)/301(20 h). P values from one-way Anova with Kruskal-Wallis multiple comparisons. (**d**) Frequency distribution of signal intensities of $Nur77^{GFP}$ expression levels in the absence of OVAp. Threshold for T cell activation was set and is indicated in the graph. (**e**) Gaussian line fitted to frequency distribution of $Nur77^{GFP}$ signal intensities in the absence or presence of OVAp and in dependence of DC centriole numbers. (**a**,**b**) All images represent maximum z-projections. Source data are provided as a Source Data file. w/o: without.

Supplementary Figure 6

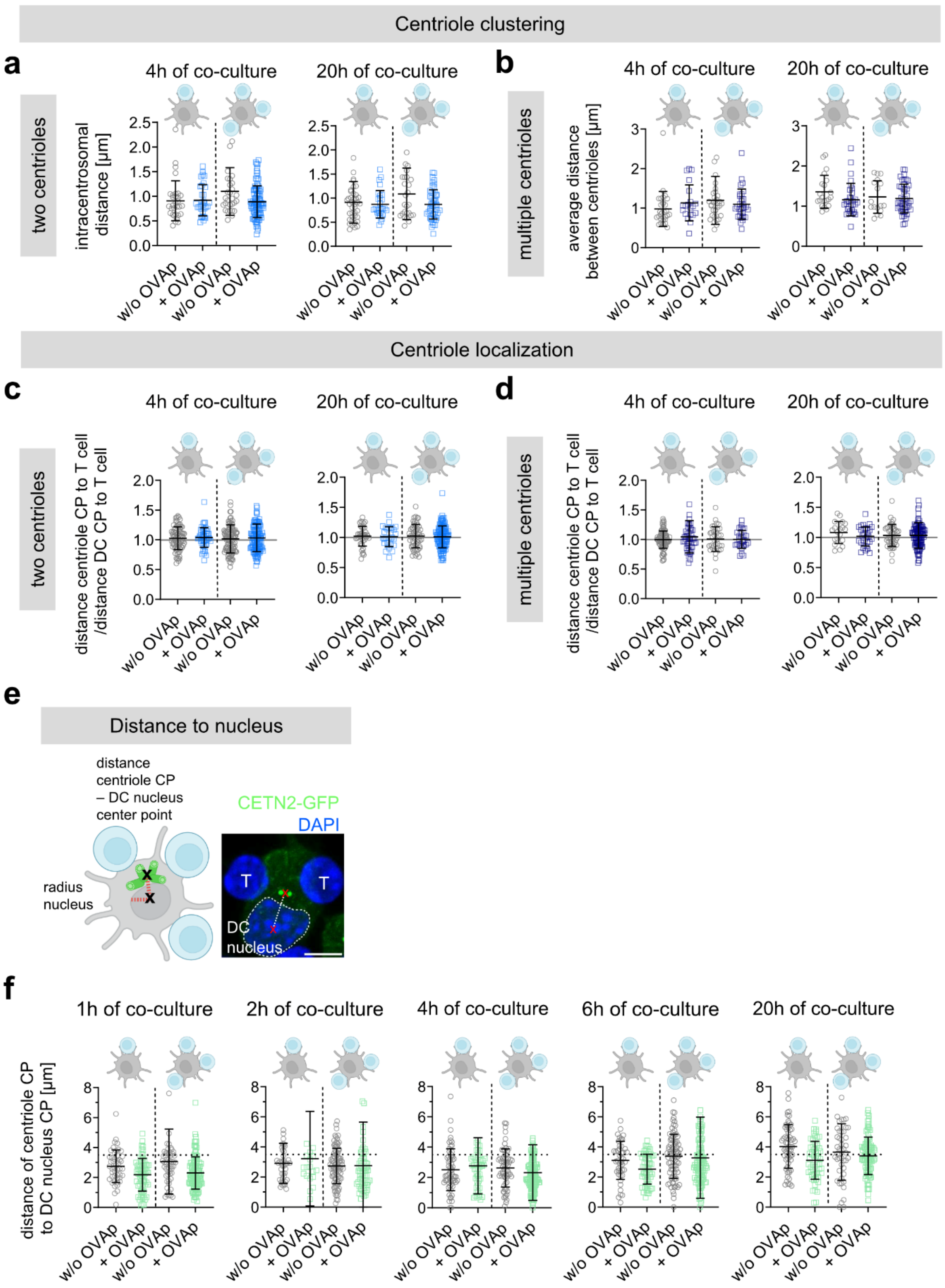

Supplementary Fig. 6.

Centriole configuration during antigen-specific DC-T cell contacts. (**a**+**b**) Quantification of intracentrosomal distances (**a**) and average distances (**b**) in DCs with two and multiple centrioles at different time points of co-culture and bound to one or several T cells (separated by dashed line and indicated on top). Graphs display mean values ± s.d. Each data point represents one cell derived from n = 5/3 (4h/20h) independent experiments. (**c**+**d**) Quantification of ratio between distance from centriole CP to T cell CP and distance DC CP to T cell in cells with two (**c**) and multiple (**d**) centrioles. Graphs show mean values ± s.d. for DCs attached to one T cell and multiple T cells as indicated on top. Each data point represents one cell derived from n = 5/3 (4h/20h) independent experiments. (**e**) Sketch indicating distance of centriole CP to center of nucleus and radius of the nucleus (red lines). Scale bar, 5 μm. (**f**) Quantification of centriole positioning relative to the nucleus displayed by distance of centriole CP to CP of the DC nucleus. Graphs represent mean values ± s.d. Each data point depicts one cell derived from n = 5/5/5/3/3 (1h/2h/4h/6h/20h) independent experiments. Dotted lines indicate radius of the nucleus. Source data are provided as a Source Data file. (**a**-**f**) Schematic pictures created with BioRender. CP: center point, w/o: without.

Supplementary Figure 7

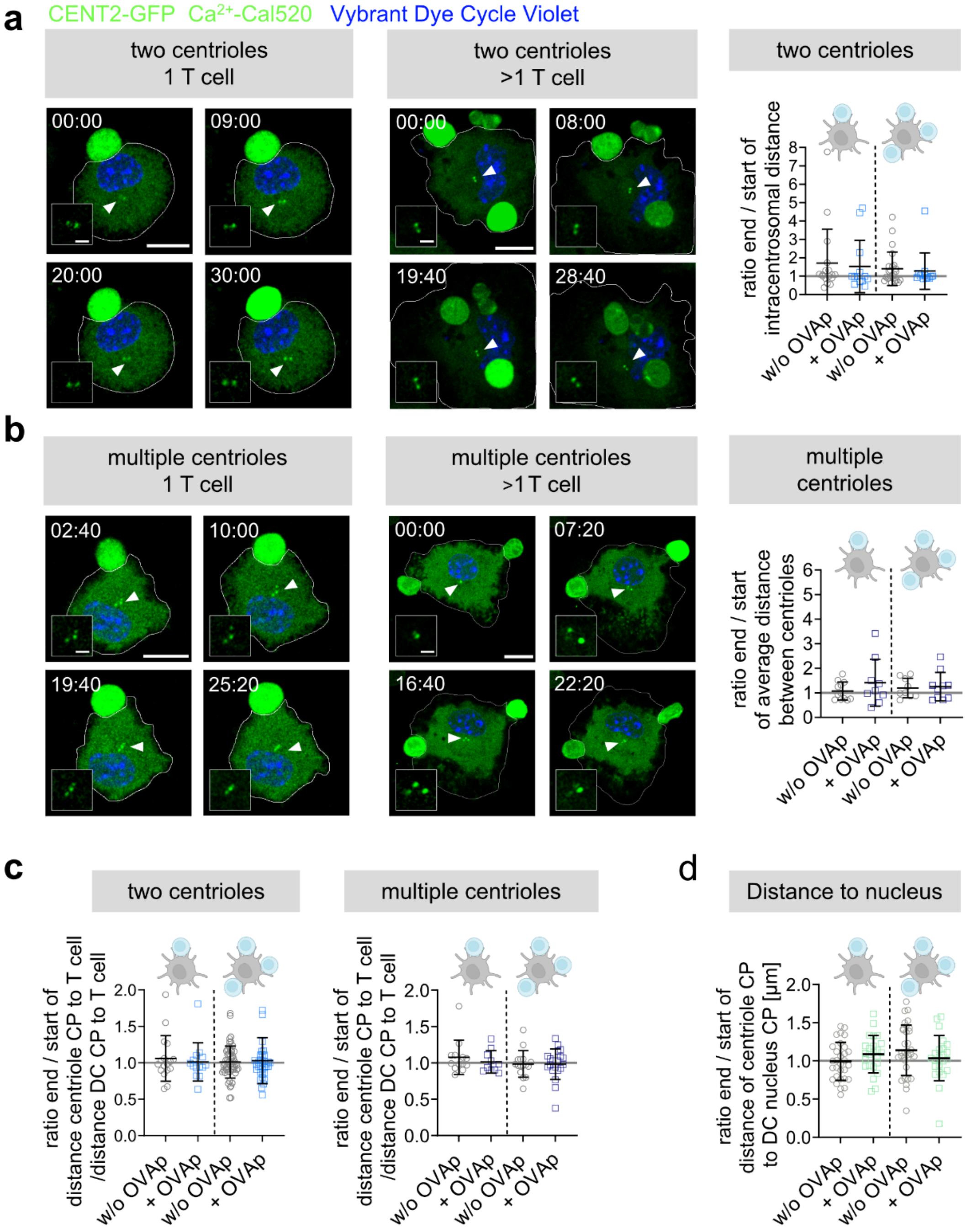

Supplementary Fig. 7.

Live cell imaging of centriole dynamics at the IS. (**a+b**) Time-lapse live-cell confocal microscopy of antigen-specific DC-T cell contacts. Left: merged images of CETN2-GFP (green), Ca^{2+}-Cal520 (green) and DNA stain (Vybrant Dye Cycle Violet, blue) are shown. Frames were collected every 20 s. Only selected frames are shown in montage with precise time points indicated in min:sec. White arrow heads point to position of centrioles. Scale bars, 10 µm. Insets show magnifications of centrioles. Scale bars, 2 µm. (**a**) Right: quantification of ratio of end vs. start intracentrosomal distance in cells with only two centrioles and dividing mono-conjugated (left half) and multi-conjugated synapses (right half). Graph shows mean values ± s.d. Each data point represents one cell recorded for at least 30-60 min from *n* = 7/6/9/6 (left to right) independent experiments. (**b**) Right: quantification of ratio of end vs. start average distance between centrioles in cells with multiple centrioles and dividing mono-conjugated (left half) and multi-conjugated synapses (right half). Graph shows mean values ± s.d. Each data point represents one cell recorded for 30-60 min from *n* = 7/4/6/4 (left to right) independent experiments. (**c**) Quantification of ratio of end vs. start of distance centriole CP to T cell CP normalized to the movement of the DC. Left (light blue) graph shows DCs with two centrioles. Right (dark blue) graph shows cells with >2 centrioles. Graphs show mean values ± s.d. Each data point represents one cell recorded for 30-60 min from *n* = 7/6/8/6 (left graph: left to right) or *n* = 7/4/6/4 (right graph: left to right) independent experiments. (**d**) Quantification of ratio of distances between centriole CP and the center of the DC nucleus at the end vs. the beginning of recording. Graphs represent mean values ± s.d. Each data point depicts one cell derived from *n* = 11/9/9/7 (left to right) independent experiments. Source data are provided as a Source Data file. (**a-d**) Schematic pictures created with BioRender. CP: center point, w/o: without.

Supplementary Figure 8

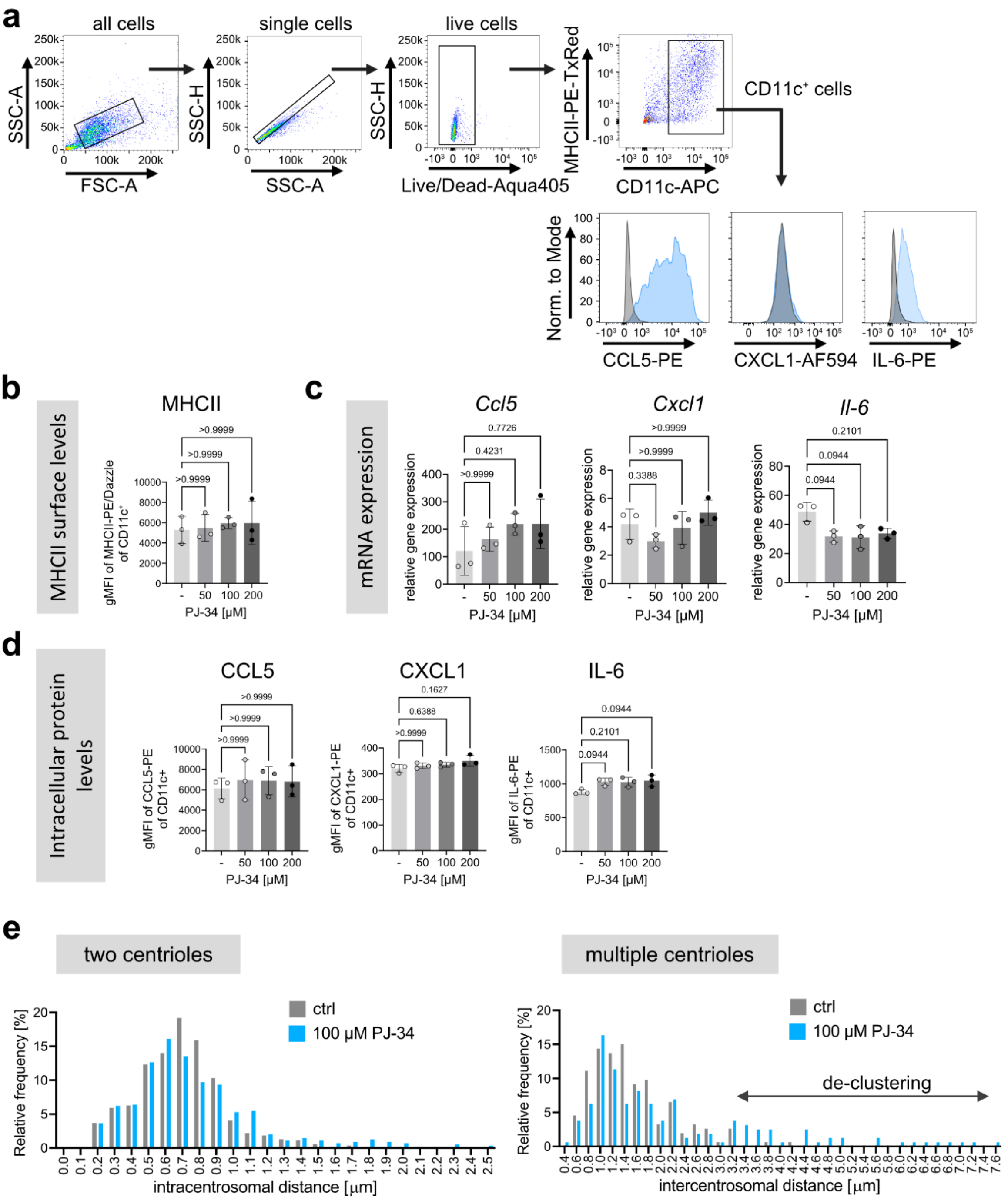

Supplementary Fig. 8.

T cell activation in the presence of the de-clustering agent PJ-34. (**a**) Gating strategy for analysing MHCII surface levels and intracellular cytokine levels of BMDCs treated with PJ-34. Grey filled lines represent unstained samples. (**b**) Graph shows geometric mean fluorescence intensity (gMFI) of MHCII in CD11c$^+$ DCs. Each data point represents one independent experiment measured in duplicates with N = 10.000 cells analyzed per condition. (**c**) Graphs show relative gene expression of *Ccl5*, *Cxcl1* and *Il-6* normalized to the house keeping gene TATA-binding protein. Each data point represents one independent experiment measured in duplicates. (**d**) Graphs show gMFI of CCL5, CXCL1 and IL-6 in CD11c$^+$ DCs. Each data point represents one independent experiment measured in duplicates with N = 10.000 cells analyzed per condition. (**b-d**) P values from Kruskal-Wallis test with Dunn's multiple comparisons. (**e**) Frequency distribution of intracentrosomal (left) and intercentrosomal (right) distances in mature CETN2-GFP expressing DCs after PJ-34 or control treatment. Cells derived from three independent experiments. Left: N = 542 (ctrl) /558 (PJ-34); Right: N = 153 (ctrl) /159 (PJ-34). Source data are provided as a Source Data file. ctrl: control, Norm.: normalized.

Supplementary Figure 9

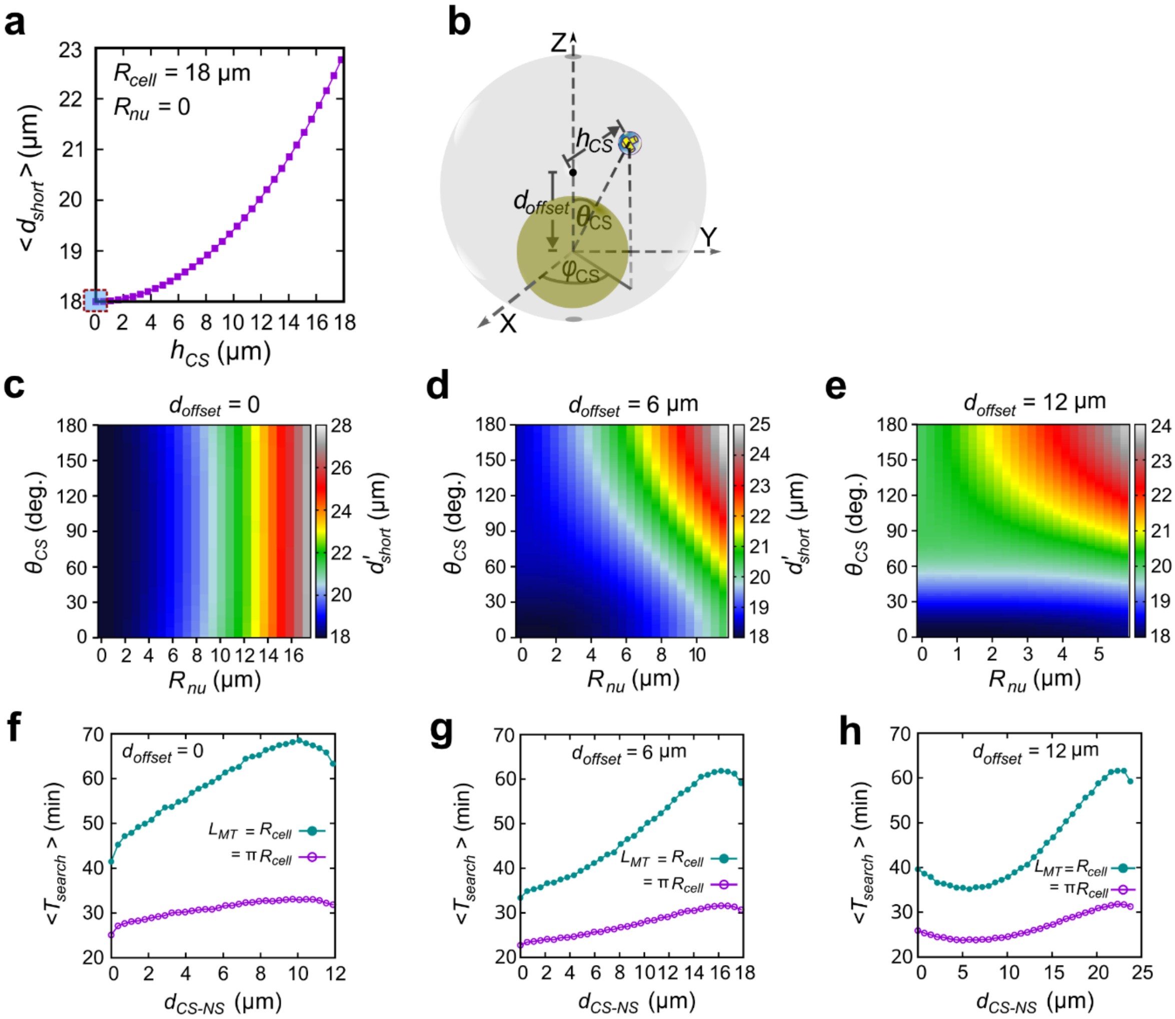

Supplementary Fig. 9.

Modelling optimal centrosome position and configuration in DCs. (**a**) Geometrically optimal centrosome position in the absence of a nucleus. Plot of the average shortest distance between the centrosome and the target points on the cell surface, $< d_{short} >$, as a function of the centrosome distance from the cell center, h_{CS}. The red outlined box denotes the optimized value of $< d_{short} >$ when the centrosome is placed at the cell center, as obtained from both simulations and our analytical calculations in *Supplementary Methods.* (**b-e**) Centrosome placed along the line joining the cell and nucleus center gives the global minimum in the average shortest distance between the centrosome and the target points on the cell surface. (**b**) A schematic depiction of the centrosome position denoted by the polar angle θ_{CS} and the azimuthal angle ϕ_{CS}. The values of $< d_{short} >$ involving isotropic target points on the cell surface do not depend on ϕ_{CS}, due to the spherical symmetry of both the cell and the nucleus in the model. (**c-e**) Values of d'_{short} plotted as a function of θ_{CS} and nuclear radius, R_{nu}, for different off-centered positions of the nucleus. d'_{short} represents the optimal value of $< d_{short} >$ obtained by varying the centrosome's position at different distances from the nucleus while keeping the θ_{CS} fixed. (**f-h**) Average search times dependent on average MT length. The average search time, $< T_{search} >$, varies with centrosome position away from nuclear surface, d_{CS-NS}, showing qualitatively similar behavior for different average MT lengths, albeit with comparatively smaller search time for MTs having larger average length. (**b**) Scheme was custom-drawn using Inkscape (v0.92.5). Source data are provided as a Source Data file.

Supplementary Figure 10

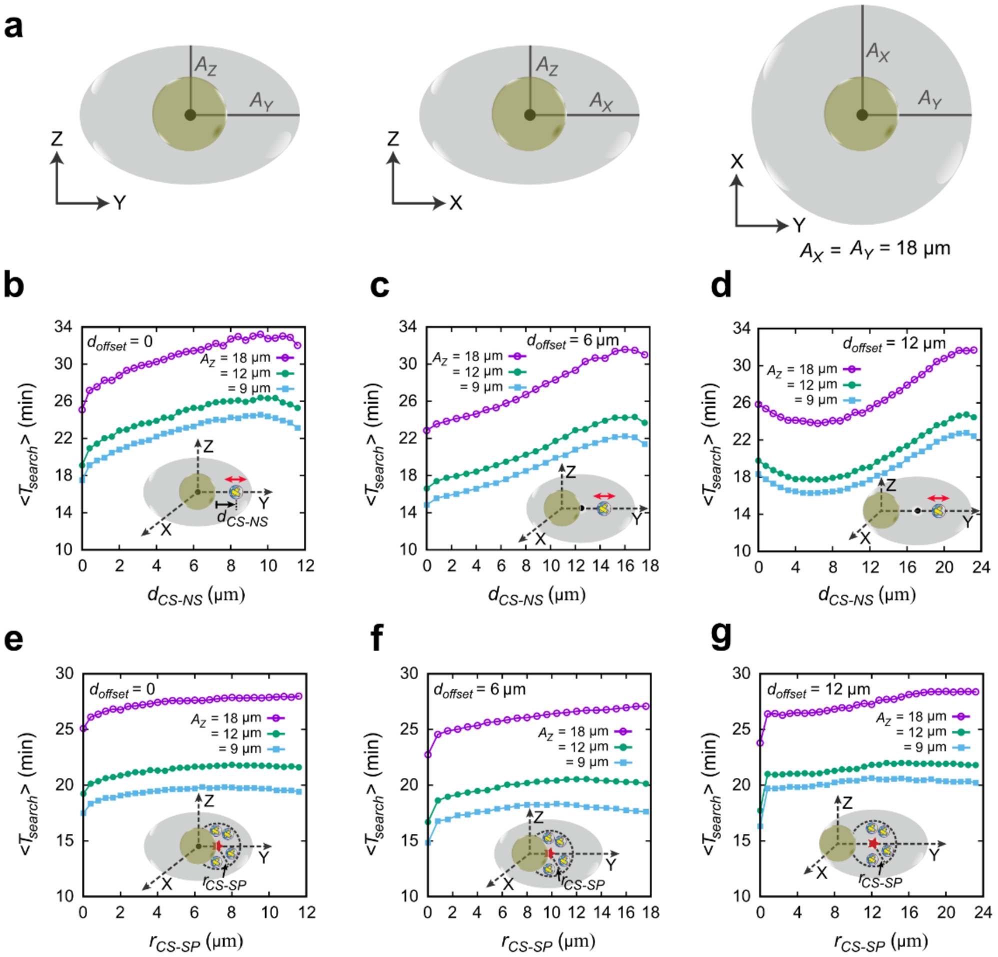

Supplementary Fig. 10.

T cell priming in flattened cells exhibits similar optimization features as in rounded cells. (**a**) The cell is flattened along the z-axis, as illustrated by the corresponding YZ, XZ, and XY views. The semi-axes of the cell are denoted by $A_X, A_Y,$ and $A_Z,$ where $A_X = A_Y = A_Z$ corresponds to a spherical cell, and flattening is introduced by setting $A_Z < A_X (= A_Y)$. (**b-d**) Plots of average search time ($< T_{search} >$) versus the distance between the centrosome and the nuclear surface (d_{CS-NS}) for different off-centered nuclear positions and three cell geometries: $A_z = 18$ µm (spherical), 12 µm, and 9 µm (flattened). For simplicity, both the nucleus and centrosome are displaced along the long axis of the cell (see insets). (**e-g**) Plots of $< T_{search} >$ vs r_{CS-SP} for different nuclear offsets and $A_z = 18$ µm (spherical), 12 µm, and 9 µm (flattened), respectively. r_{CS-SP} is the radius of the imaginary sphere centered at the optimal centrosome position (red star in insets) obtained in panels (b-d), within which centrosomes are randomly distributed. In all cases, microtubules are not allowed to glide along the cell surface. (**a**) Schemes were custom-drawn using Inkscape (v0.92.5). Source data are provided as a Source Data file.

Supplementary Figure 11

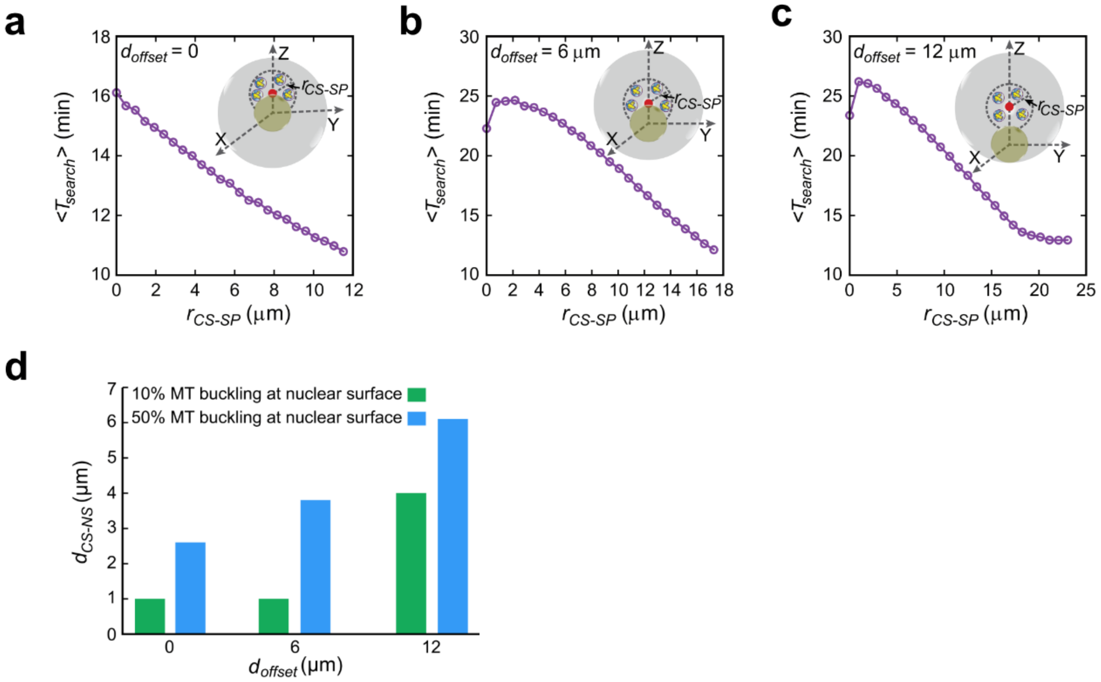

Supplementary Fig. 11.

Model predictions of search time with dispersed centrosomes (or centrioles) and freely gliding MTs along the cell surface, and a mechanistic model regulating the positioning of closely placed centrosomes (centriole cluster). (**a-c**) Dispersed centrosomes reduce the search time in the presence of unrestricted MTs gliding along the cell surface. Average search time, $< \boldsymbol{T_{search}} >$, is plotted against $\boldsymbol{r_{CS-SP}}$ for different off-centered positions of the nucleus. $\boldsymbol{r_{CS-SP}}$ is the radius of the imaginary sphere centered around a specifically chosen point (red) inside the cell (the points are considered perinuclear for $\boldsymbol{d_{offset}}$= 0 and at the cell center for $\boldsymbol{d_{offset}}$= 6 µm and 12 µm), within which centrosomes are randomly placed, as shown schematically in the insets of the figures. (**d**) The mechanistic force-balance model demonstrates the rise in the distance between the centriole cluster (centroid of the centrosomal aggregate) and the nuclear surface when a higher fraction of MTs is assumed to buckle at the nuclear surface. The data in the plot is compared for scenarios where either 10% or 50% of the MTs hitting the nucleus buckle at the nuclear surface. **(a-c)** Schemes were custom-drawn using Inkscape (v0.92.5). Source data are provided as a Source Data file.

	Supplementary Table 1: List of parameters	
**Abbreviations**	**Meaning**	**Value | Range | Reference**
R_{cell}	Cell radius	18 μm |[4]
R_{nu}	Nucleus radius	$R_{cell}/3$ |[4]
d_{offset}	Distance between the nucleus center and cell center	0, 6 μm, and 12 μm | This study
R_{τ}	Target radius	2 μm |[3]
N_{MT}	Total number of MTs	40 |[4]
L_{MT}	Average MT length	πR_{cell} | $R_{cell} - \pi R_{cell}$ | This study
v_g	MTs growth velocity	14.3 μm/min |[1,2]
v_s	MTs shrinkage velocity	16 μm/min |[1,2]
f_c	MTs catastrophe frequency	v_g/L_{MT} |[5]
f_r	MTs rescue frequency	0 |[1,2,6]
k_0, α_d	The phenomenological constant determining the sensitivity of dissociation of nuclear gliding MTs	0.1, $\pi/2$ radian | This study*
λ_l	The phenomenological constant determining the increasing rate of catastrophe frequency of cell surface-gliding MTs	$0.1 - 10$ μm^{-1} | This study
η	Co-efficient of cytoplasmic viscosity	~200 pN s μm^{-2} |[7,8]

Supplementary Table 1

Parameters used in the computational model to simulate centrosomal arrangements in DCs.

* The value of $\boldsymbol{k_0}$ and $\boldsymbol{\alpha_d}$ in Supplementary Table 1 are chosen in a manner such that they ensure the successful capture of targets across a broad range of positions that are not directly visible to MTs from the centrosomal location due to nuclear hindrance. A larger $\boldsymbol{k_0}$ values and/or smaller $\boldsymbol{\alpha_d}$ values may result in faster MT dissociation, potentially leaving targets positioned well below the equatorial plane (hiding far below the nucleus) uncaptured. Conversely, a smaller $\boldsymbol{k_0}$ values and/or larger $\boldsymbol{\alpha_d}$ values encourage MTs to continue gliding along the nuclear surface, delaying their dissociation. This can pose challenges when capturing targets that have just become directly inaccessible to MTs due to nuclear hindrance. Therefore, intermediate values of $\boldsymbol{k_0}$ and $\boldsymbol{\alpha_d}$ as used in Supplementary Table 1 can ensure the target capture for all positioning of target for which the direct capture is not possible.

SI References

1. Sarkar, A., Rieger, H. & Paul, R. Search and Capture Efficiency of Dynamic Microtubules for Centrosome Relocation during IS Formation. *Biophys. J.* **116**, 2079–2091 (2019).

2. Holy, T. E. & Leibler, S. Dynamic instability of microtubules as an efficient way to search in space. *Proc. Natl. Acad. Sci.* **91**, 5682–5685 (1994).

3. Brossard, C. *et al.* Multifocal structure of the T cell – dendritic cell synapse. *Eur. J. Immunol.* **35**, 1741–1753 (2005).

4. Weier, A.-K. *et al.* Multiple centrosomes enhance migration and immune cell effector functions of mature dendritic cells. *J. Cell Biol.* **221**, e202107134 (2022).

5. Verde, F., Dogterom, M., Stelzer, E., Karsenti, E. & Leibler, S. Control of microtubule dynamics and length by cyclin A- and cyclin B-dependent kinases in Xenopus egg extracts. *J. cell Biol.* **118**, 1097–1108 (1992).

6. Wollman, R. *et al.* Efficient Chromosome Capture Requires a Bias in the 'Search-and-Capture' Process during Mitotic-Spindle Assembly. *Curr. Biol.* **15**, 828–832 (2005).

7. Letort, G., Nedelec, F., Blanchoin, L. & Théry, M. Centrosome centering and decentering by microtubule network rearrangement. *Mol. Biol. Cell* **27**, 2833–2843 (2016).

8. Som, S., Chatterjee, S. & Paul, R. Mechanistic three-dimensional model to study centrosome positioning in the interphase cell. *Phys. Rev. E* **99**, 012409 (2019).
