# Supplementary material for: A centrally positioned cluster of multiple centrioles in antigen-presenting cells fosters T cell activation
Source: Nat Commun. 2026 Jan 13;17:536. doi: 10.1038/s41467-026-68286-7 (PMC12804990; doi:10.1038/s41467-026-68286-7)
Supplement: Supplementary file 2 — Description of Additional Supplementary Files [file 41467_2026_68286_MOESM2_ESM.pdf]

Supplementary movie 1
Live cell imaging of centriole dynamics during immune synapse formation
Time-lapse live-cell confocal microscopy of antigen-specific DC-T cell contacts. Merged channels of CETN2-GFP (green), Ca^{2+}-Cal520 (green) and DNA stain (Vybrant Dye Cycle Violet, blue) are displayed. Movie shows DC carrying two centrioles and forming a mono-conjugated synapse. White arrow heads point to the position of individual centrioles. Cell-cell contacts were imaged with frame rate of 20 s. Played as 10 frames/s. Scalebar 10 µm.

Supplementary movie 2
Live cell imaging of centriole dynamics during immune synapse formation
Time-lapse live-cell confocal microscopy of antigen-specific DC-T cell contacts. Merged channels of CETN2-GFP (green), Ca^{2+}-Cal520 (green) and DNA stain (Vybrant Dye Cycle Violet, blue) are displayed. Movie shows DC carrying two centrioles and forming a multi-conjugated synapse. White arrow heads point to the position of individual centrioles. Cell-cell contacts were imaged with frame rate of 20 s. Played as 10 frames/s. Scalebar 10 µm.

Supplementary movie 3
Live cell imaging of centriole dynamics during immune synapse formation
Time-lapse live-cell confocal microscopy of antigen-specific DC-T cell contacts. Merged channels of CETN2-GFP (green), Ca^{2+}-Cal520 (green) and DNA stain (Vybrant Dye Cycle Violet, blue) are displayed. Movie shows DC carrying multiple centrioles and forming a mono-conjugated synapse. White arrow heads point to the position of individual centrioles. Cell-cell contacts were imaged with frame rate of 20 s. Played as 10 frames/s. Scalebar 10 µm.

Supplementary movie 4
Live cell imaging of centriole dynamics during immune synapse formation
Time-lapse live-cell confocal microscopy of antigen-specific DC-T cell contacts. Merged channels of CETN2-GFP (green), Ca^{2+}-Cal520 (green) and DNA stain (Vybrant Dye Cycle Violet, blue) are displayed. Movie shows DC carrying multiple centrioles and forming a multi-conjugated synapse. White arrow heads point to the position of individual centrioles. Cell-cell contacts were imaged with frame rate of 20 s. Played as 10 frames/s. Scalebar 10 µm.

Supplementary movie 5
Antigen-specific DC-T cell contacts in LNs
Video montage showing 3D rendering of multi-z-plane images from murine LNs visualizing DC (green), DC nucleus (blue), DC centrioles (yellow) and OT-II T cell (magenta). Number of DC centrioles and number of attached T cells are indicated at the top left corner. Scale bars, 3 µm.

Supplementary movie 6
Simulation movie demonstrating the dynamics of four closely placed centrosomes, consisting centriole aggregates, with a nucleus fixed at the cell center ($d_{offset} = 0$). The cell is depicted in gray, the MTs in blue, the nucleus in olive, and the centrosomal assembly in yellow.

Supplementary movie 7
Simulation movie demonstrating the dynamics of four closely placed centrosomes, consisting centriole aggregates, when the nucleus is off-centered such that the nuclear surface touches the cell center ($d_{offset} = 6\ \mu m$). The cell is depicted in gray, the MTs in blue, the nucleus in olive, and the centrosomal assembly in yellow.

Supplementary movie 8
Simulation movie demonstrating the dynamics of four closely placed centrosomes, consisting centriole aggregates, when the nucleus is highly off-centered such that the nuclear surface touches the cell surface ($d_{offset} = 12\ \mu m$). The cell is depicted in gray, the MTs in blue, the nucleus in olive, and the centrosomal assembly in yellow.
