# Supplementary material for: A centrally positioned cluster of multiple centrioles in antigen-presenting cells fosters T cell activation
Source: Nat Commun. 2026 Jan 13;17:536. doi: 10.1038/s41467-026-68286-7 (PMC12804990; doi:10.1038/s41467-026-68286-7)
Supplement: Supplementary file 11 — Reporting Summary [file 41467_2026_68286_MOESM11_ESM.pdf]

nature portfolio

Corresponding author(s): Eva Kiermaier and Heiko Rieger

Last updated by author(s): Dec 16, 2025

Reporting Summary

Nature Portfolio wishes to improve the reproducibility of the work that we publish. This form provides structure for consistency and transparency in reporting. For further information on Nature Portfolio policies, see our Editorial Policies and the Editorial Policy Checklist.

Statistics

For all statistical analyses, confirm that the following items are present in the figure legend, table legend, main text, or Methods section.

n/a	Confirmed	
☐	☒	The exact sample size (*n*) for each experimental group/condition, given as a discrete number and unit of measurement
☐	☒	A statement on whether measurements were taken from distinct samples or whether the same sample was measured repeatedly
☐	☒	The statistical test(s) used AND whether they are one- or two-sided<br>*Only common tests should be described solely by name; describe more complex techniques in the Methods section.*
☒	☐	A description of all covariates tested
☐	☒	A description of any assumptions or corrections, such as tests of normality and adjustment for multiple comparisons
☐	☒	A full description of the statistical parameters including central tendency (e.g. means) or other basic estimates (e.g. regression coefficient) AND variation (e.g. standard deviation) or associated estimates of uncertainty (e.g. confidence intervals)
☐	☒	For null hypothesis testing, the test statistic (e.g. *F*, *t*, *r*) with confidence intervals, effect sizes, degrees of freedom and *P* value noted<br>*Give P values as exact values whenever suitable.*
☒	☐	For Bayesian analysis, information on the choice of priors and Markov chain Monte Carlo settings
☒	☐	For hierarchical and complex designs, identification of the appropriate level for tests and full reporting of outcomes
☒	☐	Estimates of effect sizes (e.g. Cohen's *d*, Pearson's *r*), indicating how they were calculated

Our web collection on statistics for biologists contains articles on many of the points above.

Software and code

Policy information about availability of computer code

Data collection	Data was collected using the following commercial software:<br>Imaging - Laser scanning confocal microscopy (LSM880): Zen Black 2.3 SP1 (Zeiss), Spinning disc microscopy: Nikon Eclipse Ti2 microscope<br>Flow cytometry - LSR II or ARIAIII Cell Sorter with FACS Diva 6.1.3 Software (BD Bioscience)<br>Gene expression analysis - CFX96 Real-Time System (BioRad)
Data analysis	Data was analysed using the following software:<br>Microscopy data analysis - ImageJ v1.54 and IMARIS 10.2.0, Statistics - GraphPad Prism 10, Flow Cytometry - FlowJo X 10.8.1, gene expression analysis - CFX Manager Software Version 3.1 (BioRad)<br>Schematics - Inkscape v0.92.5, BioRender

For manuscripts utilizing custom algorithms or software that are central to the research but not yet described in published literature, software must be made available to editors and reviewers. We strongly encourage code deposition in a community repository (e.g. GitHub). See the Nature Portfolio guidelines for submitting code & software for further information.

Data

Policy information about availability of data

All manuscripts must include a data availability statement. This statement should provide the following information, where applicable:

- Accession codes, unique identifiers, or web links for publicly available datasets
- A description of any restrictions on data availability
- For clinical datasets or third party data, please ensure that the statement adheres to our policy

All data connected to the manuscript are available upon request.

Research involving human participants, their data, or biological material

Policy information about studies with human participants or human data. See also policy information about sex, gender (identity/presentation), and sexual orientation and race, ethnicity and racism.

Reporting on sex and gender	*Use the terms sex (biological attribute) and gender (shaped by social and cultural circumstances) carefully in order to avoid confusing both terms. Indicate if findings apply to only one sex or gender; describe whether sex and gender were considered in study design; whether sex and/or gender was determined based on self-reporting or assigned and methods used.*<br>*Provide in the source data disaggregated sex and gender data, where this information has been collected, and if consent has been obtained for sharing of individual-level data; provide overall numbers in this Reporting Summary. Please state if this information has not been collected.*<br>*Report sex- and gender-based analyses where performed, justify reasons for lack of sex- and gender-based analysis.*
Reporting on race, ethnicity, or other socially relevant groupings	*Please specify the socially constructed or socially relevant categorization variable(s) used in your manuscript and explain why they were used. Please note that such variables should not be used as proxies for other socially constructed/relevant variables (for example, race or ethnicity should not be used as a proxy for socioeconomic status).*<br>*Provide clear definitions of the relevant terms used, how they were provided (by the participants/respondents, the researchers, or third parties), and the method(s) used to classify people into the different categories (e.g. self-report, census or administrative data, social media data, etc.)*<br>*Please provide details about how you controlled for confounding variables in your analyses.*
Population characteristics	*Describe the covariate-relevant population characteristics of the human research participants (e.g. age, genotypic information, past and current diagnosis and treatment categories). If you filled out the behavioural & social sciences study design questions and have nothing to add here, write "See above."*
Recruitment	*Describe how participants were recruited. Outline any potential self-selection bias or other biases that may be present and how these are likely to impact results.*
Ethics oversight	*Identify the organization(s) that approved the study protocol.*

Note that full information on the approval of the study protocol must also be provided in the manuscript.

Field-specific reporting

Please select the one below that is the best fit for your research. If you are not sure, read the appropriate sections before making your selection.

- [x] Life sciences
- [ ] Behavioural & social sciences
- [ ] Ecological, evolutionary & environmental sciences

For a reference copy of the document with all sections, see nature.com/documents/nr-reporting-summary-flat.pdf

Life sciences study design

All studies must disclose on these points even when the disclosure is negative.

Sample size	No statistical method was used to predetermine sample size. All experiments were repeated at least three times. Individual experiments were validated separately and only pooled if showing the same trend.
Data exclusions	Data from biological replicates were excluded or not acquired if assay conditions failed to support correct experimental procedure.
Replication	Experiments were replicated at least three times whenever possible. Each experiment was run with multiple biological replicates (e.g. multiple assays per condition and experiment). Experiments were entirely independent from each other, and all replications were successful.
Randomization	Cells were allocated into individual experimental groups according to type of experiment.
Blinding	Investigators were not blinded during group allocation, data collection and analysis as the entire experiment was carried out by the same individual.

Reporting for specific materials, systems and methods

We require information from authors about some types of materials, experimental systems and methods used in many studies. Here, indicate whether each material, system or method listed is relevant to your study. If you are not sure if a list item applies to your research, read the appropriate section before selecting a response.

Materials & experimental systems

n/a	Involved in the study
☐	☒ Antibodies
☒	☐ Eukaryotic cell lines
☒	☐ Palaeontology and archaeology
☐	☒ Animals and other organisms
☒	☐ Clinical data
☒	☐ Dual use research of concern
☒	☐ Plants

Methods

n/a	Involved in the study
☒	☐ ChIP-seq
☐	☒ Flow cytometry
☒	☐ MRI-based neuroimaging

Antibodies

Antibodies used

hamster anti-mouse CD11c-PE (N418, BioLegend 117308, 1:500), rat anti-mouse MHCII (I-A/I-E)-APC-Cy7 (M5/114.15.2, BioLegend 100222, 1:800), rat anti-mouse MHCII (I-A/I-E)-eFluor450 (M5/114.15.2, Invitrogen 48-5321-82, 1:800), rat anti-mouse CD4-APC (RM4-5, BioLegend 100516, 1:500), rat anti-mouse CD19-PE (6D5, BioLegend 115507, 1:500), rat anti-mouse CD19-Pacific Blue (6D5, BioLegend 115523, 1:500), rat anti-mouse MHCII (I-A/I-E)-PE-Dazzle (M5/114.15.2, BioLegend 107648, 1:600), hamster anti-mouse CD69-FITC (H1.2F3, BioLegend 104505, 1:200), hamster anti-mouse CD69-PE-Dazzle (H1.2F3, BioLegend 104536, 1:200), rat anti-mouse CD62L-PE-Cy7 (MEL-14, BioLegend 104417 1:500), rat anti-mouse OX40 (CD134)-BV711 (OX-86, BioLegend 119421, 1:300), rat anti-mouse IL-6-PE (MP5-20F3, BioLegend 504503, 1:300), mouse anti-mouse CCL5-PE (2E9/CCL5, BioLegend 149103, 1:300), rabbit anti-mouse CXCL1-Alexa Fluor 594 (1174A, R&D Instruments IC4532T, 1:300)

rat anti-mouse alpha-tubulin (YL1/2, Invitrogen MA1-80017, 1:500), mouse anti-mouse acetylated-tubulin (C3B9, Sigma-Aldrich 00020913, 1:10), mouse anti-mouse acetylated-tubulin (6-11B-1, Sigma-Aldrich T7451, 1:500), mouse anti-mouse γ-tubulin (GTU-88, Sigma-Aldrich T6557, 1:500), rabbit anti-mouse γ-tubulin (polyclonal, Abcam AB11317, 1:500), rabbit anti-mouse CDK5RAP2 (polyclonal, Sigma-Aldrich 06-1398, 1:500), rabbit anti-mouse pericentrin (EPR21987, Abcam AB4448, 1:200), hamster anti-mouse TCRβ chain-Biotin (H57-597, BD Bioscience 553168, 1:100)

donkey Anti-Mouse Alexa Fluor 647 AffiniPure F(ab')2 Fragment IgG (H+L) (715-606-150), Donkey Anti-Mouse Cy3 AffiniPure F(ab')2 Fragment IgG (H+L)(715-166-151), Donkey Anti-Rat Cy3 AffiniPure F(ab')2 Fragment IgG (H+L)(712-165-150), Donkey Anti-Rabbit Alexa Fluor 647 AffiniPure F(ab')2 Fragment IgG (H+L)(711-606-152), Goat Anti-Rabbit Cy3 AffiniPure F(ab')2 Fragment IgG (H+L) (111-165-144), Streptavidin-Cy3 (016-160-084) (all from Jackson ImmunoResearch and used 1:400)
goat anti-rabbit Alexa Fluor 488 IgG H+L (Invitrogen A11008, 1:500), goat anti-mouse Alexa Fluor 555 IgG H+L (1:500, Invitrogen A21422)

Validation

All antibodies employed in this study were commercial antibodies purchased from indicated vendors; validation data is provided on the manufacturers website. During individual experiments, negative controls (w/o primary or secondary antibodies for immunofluorescence or flow cytometry) have been carried out to test antibody specificity.

Animals and other research organisms

Policy information about studies involving animals; ARRIVE guidelines recommended for reporting animal research, and Sex and Gender in Research

Laboratory animals

All mice employed in this study were bred on a C57BL/6J background. CETN2-GFP and Nur77-GFP mice were purchased from Jackson. OT-II mice were a gift of Sven Burgdorf. Alternatively, OT-II mice were purchased from Charles River and intercrossed with B6.SJL-Ptprca Pepcb/BoyJ (congenic CD45.1 mice, also purchased from Charles River) for one generation to obtain heterozygous OT-II CD45.1/2 mice. Female and male individuals aged between 3-5 months were used.

Wild animals

n/a

Reporting on sex

Sex was not considered in this study. In adoptive transfer experiments sexes were matched.

Field-collected samples

n/a

Ethics oversight

The study was performed according to national regulations (LAVE NRW).

Note that full information on the approval of the study protocol must also be provided in the manuscript.

Plants

Seed stocks

Report on the source of all seed stocks or other plant material used. If applicable, state the seed stock centre and catalogue number. If plant specimens were collected from the field, describe the collection location, date and sampling procedures.

Novel plant genotypes

Describe the methods by which all novel plant genotypes were produced. This includes those generated by transgenic approaches, gene editing, chemical/radiation-based mutagenesis and hybridization. For transgenic lines, describe the transformation method, the number of independent lines analyzed and the generation upon which experiments were performed. For gene-edited lines, describe the editor used, the endogenous sequence targeted for editing, the targeting guide RNA sequence (if applicable) and how the editor was applied.

Authentication

Describe any authentication procedures for each seed stock used or novel genotype generated. Describe any experiments used to assess the effect of a mutation and, where applicable, how potential secondary effects (e.g. second site T-DNA insertions, mosiacism, off-target gene editing) were examined.

Flow Cytometry

Plots

Confirm that:

- [x] The axis labels state the marker and fluorochrome used (e.g. CD4-FITC).
- [x] The axis scales are clearly visible. Include numbers along axes only for bottom left plot of group (a 'group' is an analysis of identical markers).
- [x] All plots are contour plots with outliers or pseudocolor plots.
- [x] A numerical value for number of cells or percentage (with statistics) is provided.

Methodology

Sample preparation

Single cell suspensions were counted and equal cell numbers used or alternatively all cells from one well prepared during the experiments were used. Cells were stained according to methods section provided in the manuscript. Cells were resuspended in Phosphate buffered saline (PBS) supplemented with 1% BSA and 2mM EDTA to prevent cell aggregation. Cell suspensions were filtered through a 35µm nylon mesh cell strainer cap prior to specimen loading.

Instrument

FACS Aria III, BD Biosciences; LSR II, BD Biosciences

Software

FACS Diva 6.1.3, BD Biosciences, FlowJo X 10.8.1

Cell population abundance

Post-sort fractions for diploid BMDCs >95%. Population purity was determined by post-sort measurement.

Gating strategy

Single cells were gated from whole cell population using forward and side scatter (FSC and SSC respectively). Doublets were removed based on SSC-A/SSC-H. To identify viable cells DRAQ7 (Biolegend) or Aqua Fixable Viability Kit (BioLegend) was used. Gates were defined based on unstained and fluorescent minus one (FMO) controls or based on clear separation between negative and positive populations.

- [x] Tick this box to confirm that a figure exemplifying the gating strategy is provided in the Supplementary Information.
